# Extension of the fluidics NTF-Biobank of polytraumatized patients with extracellular vesicles: improved vesicle preservation through local processing

**DOI:** 10.1007/s00068-026-03280-8

**Published:** 2026-07-21

**Authors:** Birte Weber, Liudmila Leppik, Jiaoyan Han, Nils Becker, Tong Wang, Rald V. M. Groven, Elisabeth Rosado Balmayor, Qun Zhao, Ramona Sturm, Borna Relja, Frank Hildebrand, Ingo Marzi

**Affiliations:** 1 Department of Trauma Surgery and Orthopedics, University Hospital Frankfurt, Goethe-University, Frankfurt am Main, Germany; 2 Translational and Experimental Trauma Research, Department of Trauma, Hand, Plastic and Reconstructive Surgery, University Hospital Ulm, Ulm, Germany; 3 Department of Orthopedic, Trauma- and Reconstructive Surgery, RWTH Aachen University Hospital, Aachen, Germany; 4 Experimental Orthopedic and Trauma Surgery, Department of Orthopedic, Trauma- and Reconstructive Surgery, RWTH Aachen University Hospital, Aachen, Germany

**Keywords:** Biobanking, NTF-EV-biobank, Multiple trauma, Size exclusion chromatography, Multicenter approach, Sample transport conditions

## Abstract

**Purpose:**

Extracellular vesicles (EVs) show diagnostic relevance in trauma, prompting the development of the NTF‑EV‑Biobank as an extension of the established Network Trauma Research (NTF) plasma/serum biobank. After optimizing the EV isolation and characterization standard operating procedure (SOP) at a single center, this multicenter study evaluates its transferability and examines how sample transport affects EV quality before large‑scale implementation in a nationwide biobank setting.

**Methods:**

Plasma and serum from polytrauma patients and healthy controls (n=10) were processed under the standardized NTF‑Biobank protocol and distributed to three centers. EVs were isolated using the unified NTF‑EV‑Biobank workflow, exchanged between centers, and analyzed for protein markers, particle size and concentration (NTA), total protein content, and morphology (TEM) to compare EV quality across sites.

**Results:**

Implementation of the EV biobank protocol across participating centers, as well as inter-center transport on dry ice, was successfully performed without complications. All samples arrived at the analyzing centers in a frozen state. EVs exhibited typical vesicular morphology as confirmed by transmission electron microscopy and remained *positive for CD9 and CD81*, indicating that transportation did not affect morphology or surface marker expression. However, transportation did reduce EV yield as assessed by NTA, while total protein content remained unchanged.

**Conclusion:**

Transport of isolated EVs between centers resulted in a significant reduction in EV yield, accompanied by a partial increase in particle size. Based on these findings, the storage and distribution of EVs in isolated form within the NTF-EV-Biobank were deemed unsuitable. Instead, samples should be stored and transported as plasma/serum, with EV isolation performed at the destination site.

**Supplementary Information:**

The online version contains supplementary material available at 10.1007/s00068-026-03280-8.

## Introduction

The nationwide biobank of serum and plasma collected from polytraumatized patients was initiated in 2013 by the Network Trauma Research (NTF) of the German Trauma Society (DGU). Its primary objective has been to provide biospecimens for multicenter studies aimed at the systematic evaluation of patient pathophysiology and the development of clinical strategies to improve outcomes. By integrating biomaterial with clinical data, this biobank project aims to enhance understanding of post-traumatic immune responses and to identify novel avenues for research. Its feasibility, including the “serumdatabank” tool, has been successfully demonstrated across seven trauma centers [1]. In recent years, there has been a marked increase in studies investigating EVs as mediators of intercellular communication and as potential biomarkers across a wide range of traumatic and non-traumatic conditions [2, 3]. According to the MISEV-2023 guideline, EVs are cell-derived, membrane-bound particles incapable of self-replication [4]. Their growing relevance stems from their capacity to transport bioactive cargo, including RNA [5], miRNAs [6], as well as DNA [7], and proteins [8], as well as surface molecules such as cell-specific proteins (e.g., CD42a + for platelet-derived EVs) [9, 10].

Work from our group as well as others have previously demonstrated the diagnostic potential of EVs in trauma. For example, an elevated overall EV concentration in the plasma has been associated with increased in-hospital mortality in polytraumatized patients [11, 12]. Reduced levels of exosomal miR-21-5p and elevated CD62p + EVs have been linked to an increased risk of septic complications in polytraumatized patients [13], while decreased CD44 + and CD31 + EVs have been associated with hemorrhagic shock [14].

To advance the field, the NTF research group FOR5417 decided to expand the previously established and successfully implemented NTF-Biobank with an EV-focused biobank (NTF-EV-Biobank). To this end, standardized protocols for EV isolation, storage, and management were developed [15]. Moreover, the impact of common quality issues in serum and plasma from polytrauma patients—such as hemolysis and lipemia—on subsequent EV analysis was evaluated [16]. In addition, as with original NTF-Biobank, the NTF-EV-Biobank was linked to clinical patient data and integrated into “serumdatabank” module. The present multicenter study aims to evaluate the transferability of the developed SOP and to assess the impact of sample transport on EV sample quality prior to large-scale implementation.

Ultimately, the conclusions derived from this study will be integrated into the final EV biobank protocol, representing the final step before systematic sample collection within the NTF-EV Biobank.

## Material and methods

The present multicenter study was approved by the Local Ethics Committee of the University of Frankfurt (approval ID: 89/19). Polytraumatized patients (Injury Severity Score [ISS] ≥ 16, *n* = 10) admitted to a Level 1 Trauma Center (Center 1) between January and December 2021 were included. Additionally, ten healthy controls (*n* = 10) were recruited.

Blood samples were collected upon admission to the emergency room (ER) and were centrifuged at 3500 × g for 15 min at 4 °C to isolate plasma and serum. Samples from healthy controls were processed identically. Subsequently, all plasma and serum aliquots were stored at − 80 °C at Center 1 (University Hospital Frankfurt) until transport to the other participating centers (Center 2, University Hospital Ulm and Center 3, University Hospital Aachen) for further analyses. The samples were transported on dry ice via a national postal service, with transit times not exceeding four days.

Along with the plasma and serum samples, participating centers received the NTF-EV isolation protocol [15], the EV isolation kit (Exo-Spin™ Exosome Size Exclusion Columns, Cell Guidance Systems, Cambridge, UK), and particle-free PBS required for EV isolation. This ensured that all three centers used identical materials and reagents. At each center, EV isolation was performed from 100 µL of plasma or serum by a single experienced laboratory scientist.

**Fig. 1:**
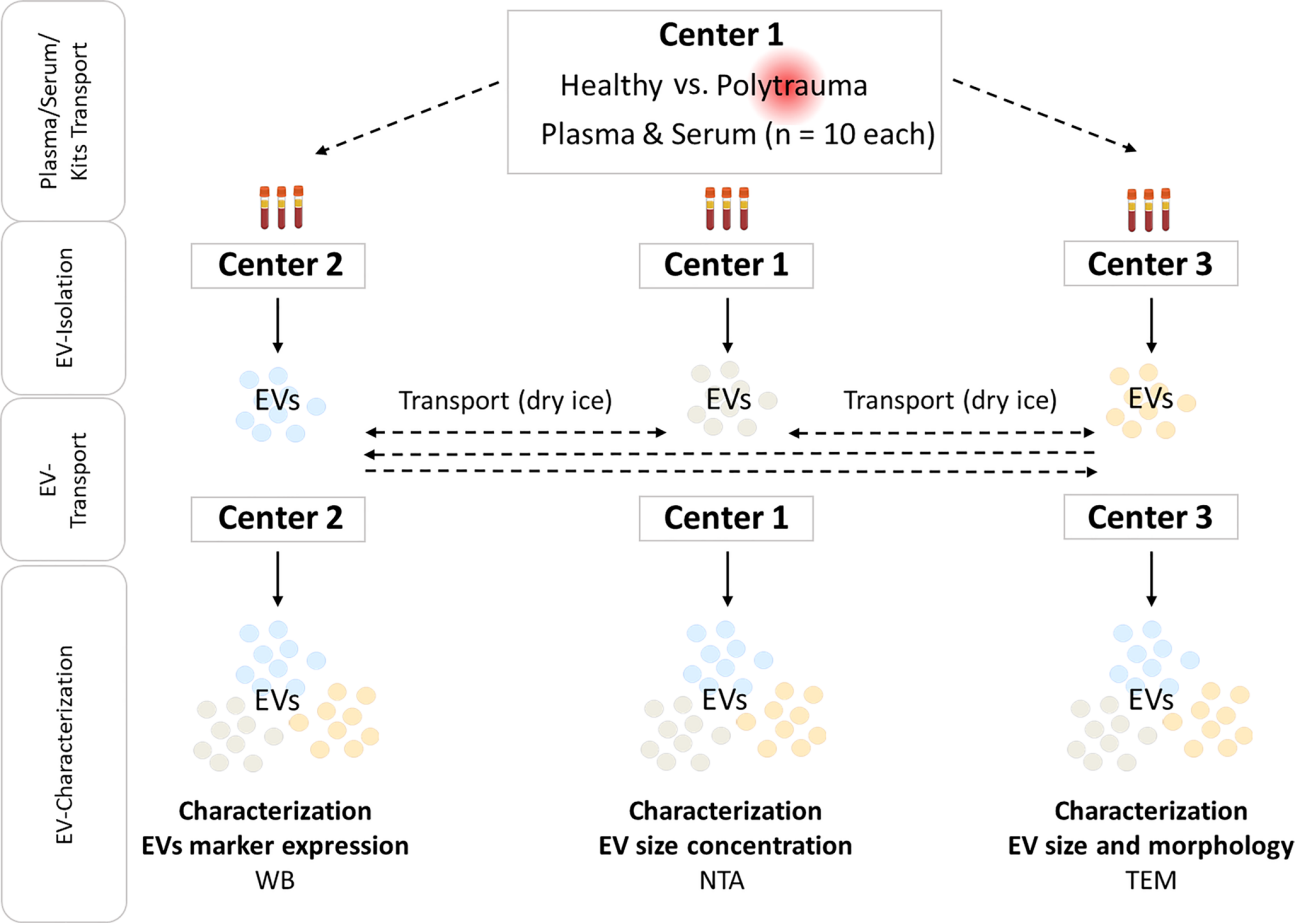
Multicenter validation study design. Plasma and serum samples were distributed from Center 1 to Centers 2 and 3 together with the isolation protocol and required kits and materials. At each center, EVs were isolated and distributed to other two centers. At each center, both locally isolated EVs as well as EVs received from the other two centers were characterized via a defined technique. EVs - Extracellular Vesicles, WB - Western Blot, NTA - Nanoparticle tracking analysis, TEM - Transmission electron microscopy. Dotted line- sample transport on dry ice

Isolated EVs were aliquoted and stored in PBS at -80 °C until further analyses or transport to the other centers for analyses. Multicenter validation was then conducted as follows: each center isolated EVs from plasma and serum samples and performed analyses specific to their center on EV isolates generated locally as well as those received from the other two centers (Fig. 1). Transportation of EV-samples was performed on dry ice via a national postal service, with transit times not exceeding four days.

### Freeze-thaw cycles

Plasma and serum samples from the biobank were thawed once to prepare aliquots for shipment to each participating center. Transport was performed on dry ice. Upon arrival, the samples were thawed once more at each center for EV isolation. The isolated EVs were subsequently frozen, transported on dry ice, and thawed only once for the respective analyses (NTA, Western blot, or TEM). Thus, all samples underwent an identical number of freeze–thaw cycles (three) and were handled under standardized conditions across centers.

### Center 1: EV particle concentration and size distribution analysis (NTA)

The concentration and size distribution of EVs were evaluated in both locally isolated EVs and those transported on dry ice from Centers 2 and 3 using nanoparticle tracking analysis (NTA). EV isolates were diluted 1:100 with 0.22 μm-filtered Dulbecco’s phosphate-buffered saline (PBS, −/−; Sigma Aldrich, Schnelldorf, Germany). Measurements were performed with a capture duration of 60 s, with six technical replicates per sample (Nanosight NS500, Malvern Panalytical, Kassel, Germany). Particles were analyzed under constant flow conditions, and the mean value of the six replicates was used for statistical analysis.

In addition, protein concentration of EV isolates was assessed via Coomassie Plus (Bradford) assay (Thermo Fisher Scientific, Rockford, IL, USA). Briefly, 10 µL of EV isolates were mixed with 300 µL of Coomassie Plus reagent and incubated for 10 min. Absorbance was measured at 595 nm, and protein concentration was calculated using a calibration curve generated from serial dilutions of a protein standard.

### Center 2: EV-marker expression analysis (Western blot)

Expression of typical EV surface-markers CD81 and CD9 was analyzed in locally isolated EV isolates as well as those transported on dry ice from the other centers. For the analyses, 4 µg of EV protein per sample were separated by electrophoresis on a 10% sodium dodecyl sulfate–polyacrylamide gel (SDS-PAGE). Proteins were subsequently transferred onto polyvinylidene fluoride (PVDF) membranes (Bio-Rad Laboratories, Hercules, CA, USA). Membranes were blocked overnight at 4 °C in blocking buffer (5% non-fat milk in TBST). Following blocking, membranes were incubated for 1 h at room temperature with primary antibodies against CD81 (1:1000, 10630D) or CD9 (1:1000, 10626D) from Invitrogen (Thermo Fisher Scientific, Waltham, MA, USA). After washing, membranes were incubated with a horseradish peroxidase (HRP)-conjugated secondary antibody (1:2000, Cell Signaling Technology, Leiden, The Netherlands) in blocking buffer for 1 h at room temperature. Protein bands were visualized using ECL™ Western Blotting Reagent (RPN2016, Merck, Taufkirchen, Germany) according to the manufacturer’s instructions.

### Center 3: EV particle size and morphology characterization (TEM)

The EV particle size and morphology of plasma EV samples, isolated locally as well as transported from the other centers, where further characterized by TEM. Briefly, 10 µl of the native EV samples were allowed to adsorb on glow discharged formvar-carbon-coated nickel grids (200 mesh, Plano, Wetzlar, Germany). Negative staining was performed after washing in distilled water with 0.5% uranyl acetate (Science Services GmbH, Munich, Germany). Samples were imaged using a Hitachi HT7800 transmission electron microscope (Hitachi, Tokyo, Japan) operating at an acceleration voltage of 100 kV. Representative images were analyzed to assess site-specific differences and the impact of the EV transport.

### Statistical analyses

All statistical analyses were performed using GraphPad Prism 9 (Dotmatics, San Diego, CA). Normality was assessed using the Kolmogorov-Smirnov test. Data are presented as mean ± standard error of the mean (SEM). For comparisons across multiple groups, the Kruskal-Walli’s test followed by Dunn’s post hoc test was applied. Pairwise group comparisons were assessed using the Mann-Whitney U test. A p-value ≤ 0.05 was considered statistically significant.

## Results

All participating centers successfully performed EV isolations without encountering any technical difficulties. Transport of EV samples between centers was completed within the targeted four-day timeframe, without complications, and all samples arrived at the receiving centers in a frozen state.

**Fig. 2:**
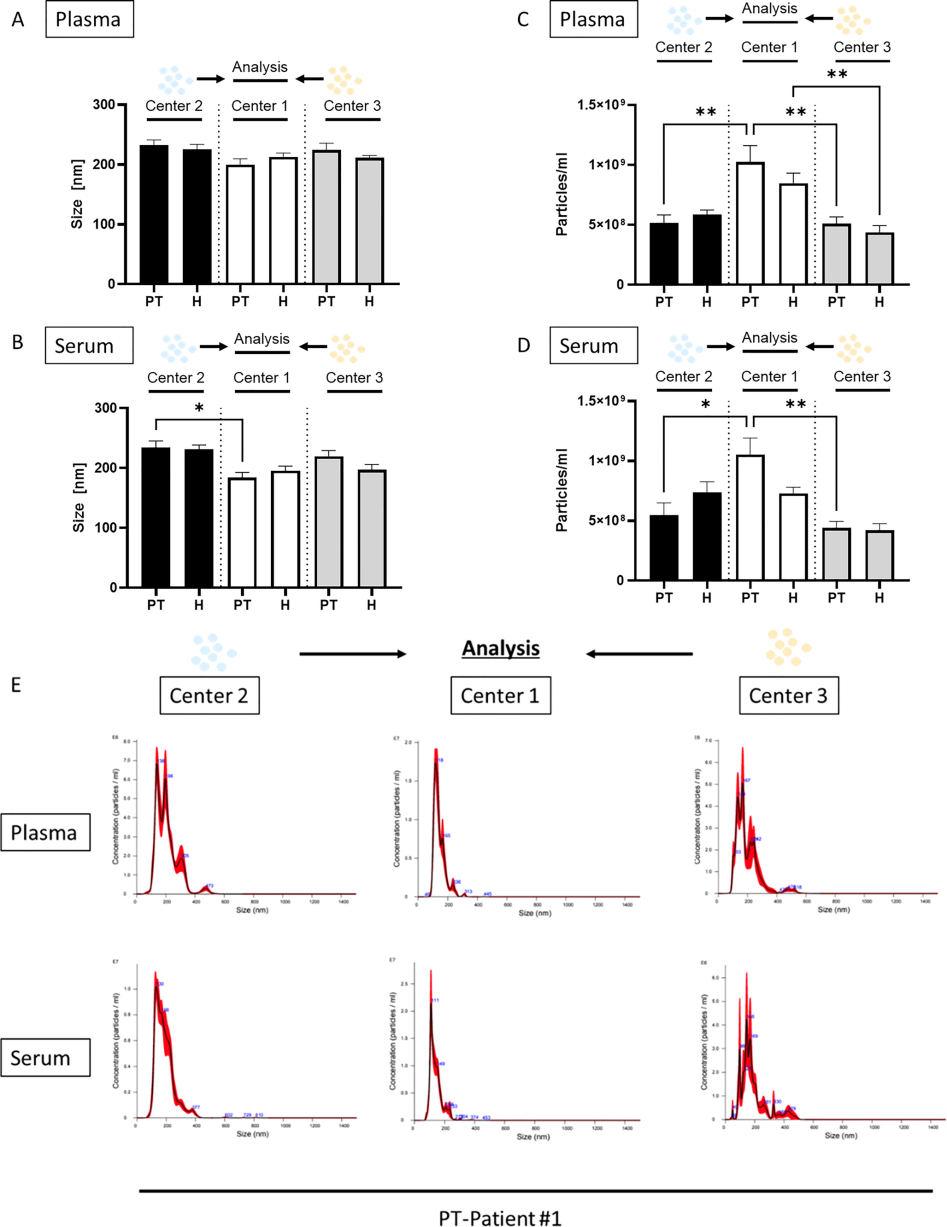
Transport-associated reduction in extracellular vesicle (EV) concentration measured by nanoparticle tracking analysis (NTA). (**A**) EVs were isolated from PT and healthy plasma, and EV particle size (nm) was analyzed at Center 1 using NTA. No significant differences in EV size were observed between locally isolated and transported EVs. (**B**) EVs were isolated from PT and healthy serum, and EV particle size (nm) was analyzed at Center 1 using NTA. Locally generated at Center 1 and transported from Center 2 PT EVs differ significantly in size. (**C**) NTA assessment of PT and healthy plasma EV concentrations (particles/ml) in samples locally generated at Center 1 or transported. Significant reduction in particle concentration after transport was observed in polytrauma EV-samples. (**D**) EVs concentration assessed in serum PT samples was also significantly reduced in transported samples. (**E**) Representative NTA of EVs isolated from plasma/serum samples of polytrauma patient no. 1, comparing locally processed samples with those transported from Centers 2 and 3. p*≤0.05; p**≤0.01, *n* = 10 each healthy (H) and polytrauma (PT), as well as serum and plasma

Particle size measurements of plasma EV samples performed at Center 1 using NTA revealed no differences among the samples (Fig. 2A). However, measurements of EVs isolated from patient serum differed compared to those isolated from healthy controls. EVs isolated at Center 2 and transported to Center 1 for analysis exhibited a significantly larger particle size compared with locally generated EVs (Fig. 2B). No difference in EV size between polytrauma patients and healthy controls was observed in either plasma or serum samples.

With respect to particle concentration, a significant reduction was observed in both PT- plasma and PT- serum EVs following transport compared with locally isolated samples (Fig. 2C and D). A similar decrease was also detected in healthy plasma EVs after transportation (Fig. 2C). NTA measurements were conducted only in Center 1 and therefor, these results are observed only after transport in one direction.

**Fig. 3:**
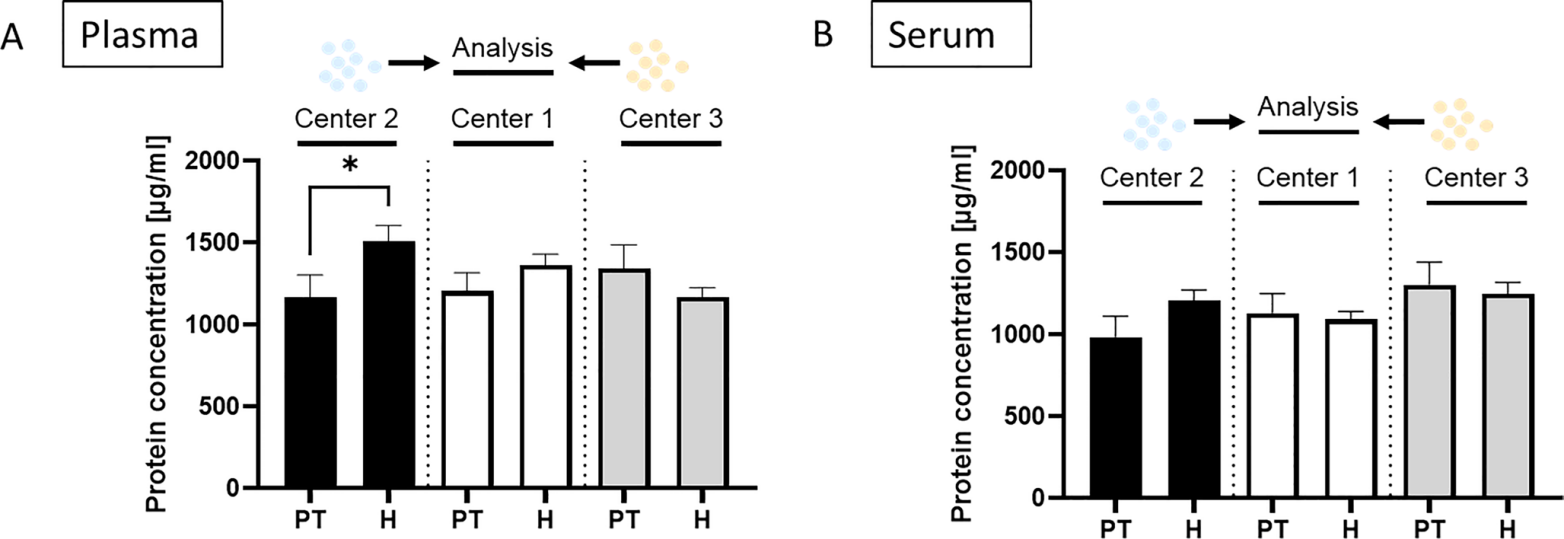
Protein concentration of extracellular vesicles (EVs) did not differ after transport. (**A**) Protein concentration of plasma-derived EVs did not differ between the three isolating centers, however there is a significant difference among PT and healthy EVs samples isolated in Center 2. (**B**) Serum-derived EVs showed no differences in protein concentration between transported and non-transported samples. p*≤0.05, *n* = 10 each healthy (H) and polytrauma (PT), as well as serum and plasma

Measurements of protein content showed no differences between transported and non-transported samples. However, plasma-derived EVs from healthy volunteers exhibited significantly higher protein concentrations compared to those from PT patients transported from Center 2 (Fig. 3A). In contrast, no differences in protein concentrations were observed between centers for serum-derived EVs (Fig. 3B).

**Fig. 4:**
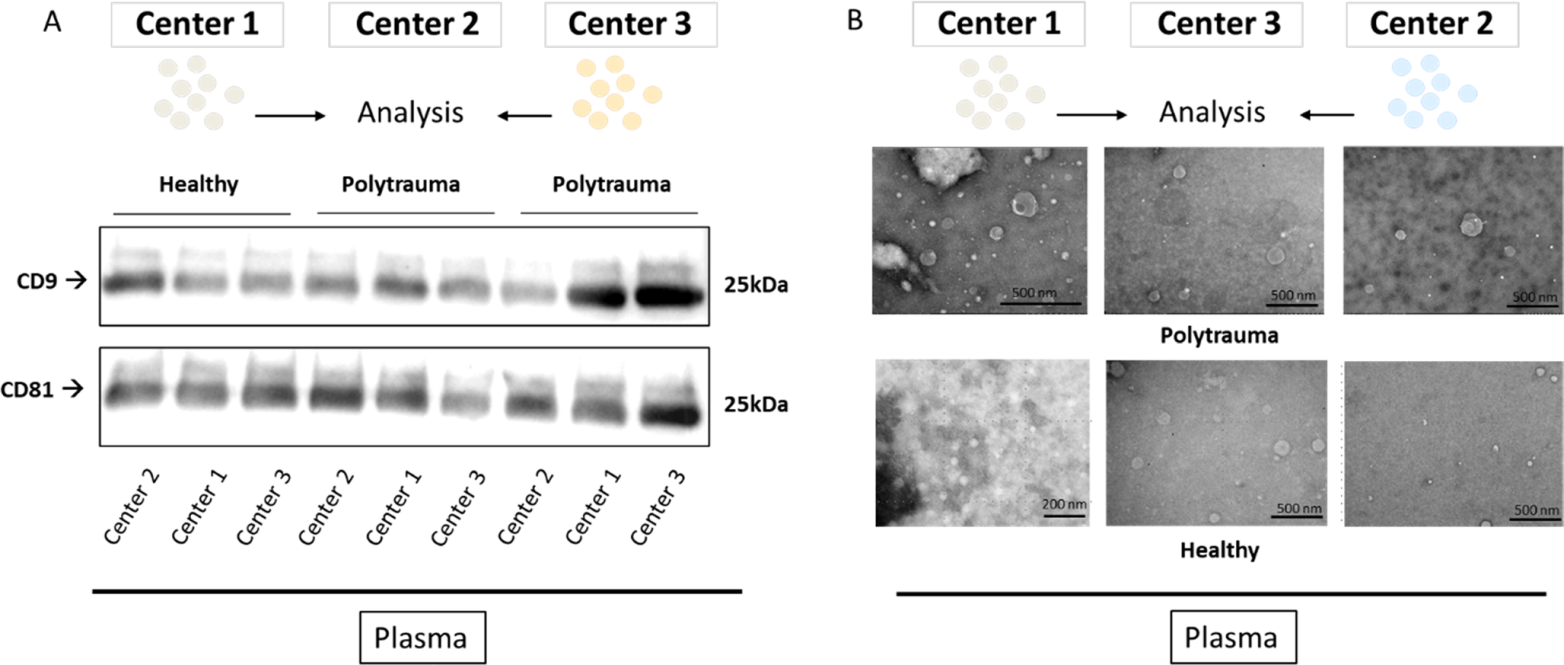
Marker expression and particle morphology of extracellular vesicles (EVs) generated locally or after transport. (**A**) Expression of typical EV markers CD9 and CD81 was shown in locally generated and transported EVs, western blot analysis of representative samples. (**B**) EV particle morphology of transported and locally generated samples, transmission electron microscopy (TEM) of representative samples

Expression of EV surface markers CD9 and CD81 was assessed by western blot from EVs either generated locally at Center 2 or transported on dry ice from Centers 1 and 3. All samples, whether transported or not, resulted positive for both CD9 and CD81 (representative results are shown in Fig. 4A).

Furthermore, the morphology and size of representative EV samples were evaluated by TEM at Center 3 (Fig. 4B). Regardless of whether the EVs had been transported, all isolates exhibited the characteristic shape and size of EVs.

## Discussion

Although EVs are considered more stable compared to whole cells, their integrity can still be affected by storage and transport conditions [17, 18]. For biobanking purposes, standardized handling, controlled storage, and careful transport are essential to preserve EV quality, maintain experimental reproducibility, and enable reliable downstream analyses across multiple laboratory sites. In a recent study, we established standardized protocols for the isolation, storage, and management of EV samples within the NTF-EV-Biobank [15]. However, the crucial step of sample transportation, which is particularly important for multicenter biobank projects, has not yet been evaluated in detail.

Considering the specific characteristics of trauma patient samples—such as their uniqueness, limited volume, and practical constraints in clinical settings, two potential strategies for a multicenter EV biobank were considered. One approach involves collecting EV samples at participating centers, shipping them to the NTF-EV-Biobank for storage, and subsequently distributing them to other sites for analysis. The alternative strategy, comparable to that routinely used in the NTF-Biobank, is to collect plasma or serum samples at participating centers, ship and store extra aliquots at the NTF-EV-Biobank, and then distribute them to the study center, where EVs can be isolated locally according to the standardized NTF-EV-Biobank SOP. Therefore, the present multicenter study had a twofold aim: to evaluate the impact of EV sample transportation between participating biobank centers and to assess the applicability of the developed SOP at these centers, to finalize the future format of the NTF-EV-Biobank.

The results of the present study showed that the implementation of the NTF-EV-Biobank SOP (which was developed in Center 1) did not encounter any technical, time-related, or personnel-related issues at the two other centers. But the transportation of EV isolates between the centers significantly affected sample quality, particularly for trauma patient samples. Specifically, we observed a notable reduction in the number of EV particles following transport, whereas protein concentration, particle size, and morphology remained mostly unchanged, suggesting that some particles were likely disrupted during transportation. In our previous study, we observed similar effects when EV isolates were stored on dry ice for five days [15]. Considering that transportation via postal service may introduce additional stressors, such as shaking or inversion, it is understandable that even a shorter duration on dry ice during shipment could have a stronger impact on sample quality.

Other authors have shown that freeze–thaw cycles reduce EV particle concentration and increase particle size, likely due to fusion or aggregation [19]. In the present study, the number of freeze–thaw cycles was kept consistent across all centers. Although overall transport conditions were well controlled, transport-related factors may still have contributed to similar effects, potentially explaining the observed increase in particle size in serum-derived EV samples isolated at Center 2 after transport. In addition, EV surface markers are known to be sensitive to temperature changes [20, 21]. While all EV isolates in our study were positive for CD9 and CD81, this assessment was qualitative and limited to only two markers and therefore does not exclude potential alterations in other surface markers. Furthermore, EV morphology is also temperature-sensitive, with vesicle enlargement, fusion, and membrane deformation reported after long-term storage at 4 °C (21). TEM imaging did not indicate any clear changes in EV morphology, although subtle differences may not have been fully captured.

Importantly, our results suggest that EVs derived from trauma patient samples may be more susceptible to transport-related effects. This is likely influenced by fundamental differences in the blood milieu between polytrauma and healthy controls. Trauma induces a systemic inflammatory response and tissue injury, leading to increased circulating proteases, such as neutrophil elastase [22], matrix metalloproteinases [23], and nucleases, which may affect EV surface proteins, membrane integrity, and cargo [24]. In addition, polytrauma-associated oxidative stress [25, 26], pH alterations [17, 27], and platelet activation may further compromise EV stability, promoting aggregation, fusion, or degradation [19, 28]. Taken together, these observations suggest that EVs in polytrauma patients may exist within a pathologically altered blood environment that could increase their vulnerability to transport-, freeze–thaw-, and storage‑related stress. In contrast, EVs from healthy donors appear comparatively more stable under similar conditions. However, these interpretations remain hypothetical, and further dedicated studies will be required to systematically investigate the underlying mechanisms and validate these proposed explanations.

In general, these findings indicate that, of the two strategies initially considered, the first—isolating EVs at participating centers, shipping them to the NTF-EV-Biobank for storage, and then distributing them to other sites—is not optimal for polytrauma patient samples, as it may compromise sample quality due to transportation. Reports in the literature indicate that EVs exhibit greater stability in their native biofluids, such as plasma or serum, than when isolated and suspended in buffer solutions [29]. This observation supports the strategy of collecting, storing, and shipping plasma or serum samples within the biobank, with EV isolation performed at the research center immediately prior to further analysis.

In summary, for the NTF-EV-Biobank, both patient and healthy samples should be stored and transported as plasma or serum, with EV isolation performed on-site in the research laboratory according to the standardized NTF-EV-Biobank protocol to ensure optimal sample quality and reliability.

## Conclusion

Transport of isolated EVs between centers resulted in a significant reduction in EV yield, accompanied by a partial increase in particle size. Notably, patient-derived EVs were more sensitive to the effects of transportation. Based on these findings, the storage and distribution of EVs in isolated form within the NTF-EV-Biobank were deemed unsuitable. Instead, samples should be stored and transported as plasma or serum, with EV isolation performed at the destination site in accordance with the standardized NTF-EV-Biobank protocol to ensure optimal sample integrity and analytical reliability.

## Supplementary Information

Below is the link to the electronic supplementary material.

Supplementary Material 1

Supplementary Material 2

## Data Availability

All data supporting the findings of this study are available within the paper.
