# Supplementary figures and images for: Extension of the fluidics NTF-Biobank of polytraumatized patients with extracellular vesicles: improved vesicle preservation through local processing
Source: Eur J Trauma Emerg Surg. 2026 Jul 21;52(1):226. doi: 10.1007/s00068-026-03280-8 (PMC13388434; doi:10.1007/s00068-026-03280-8)

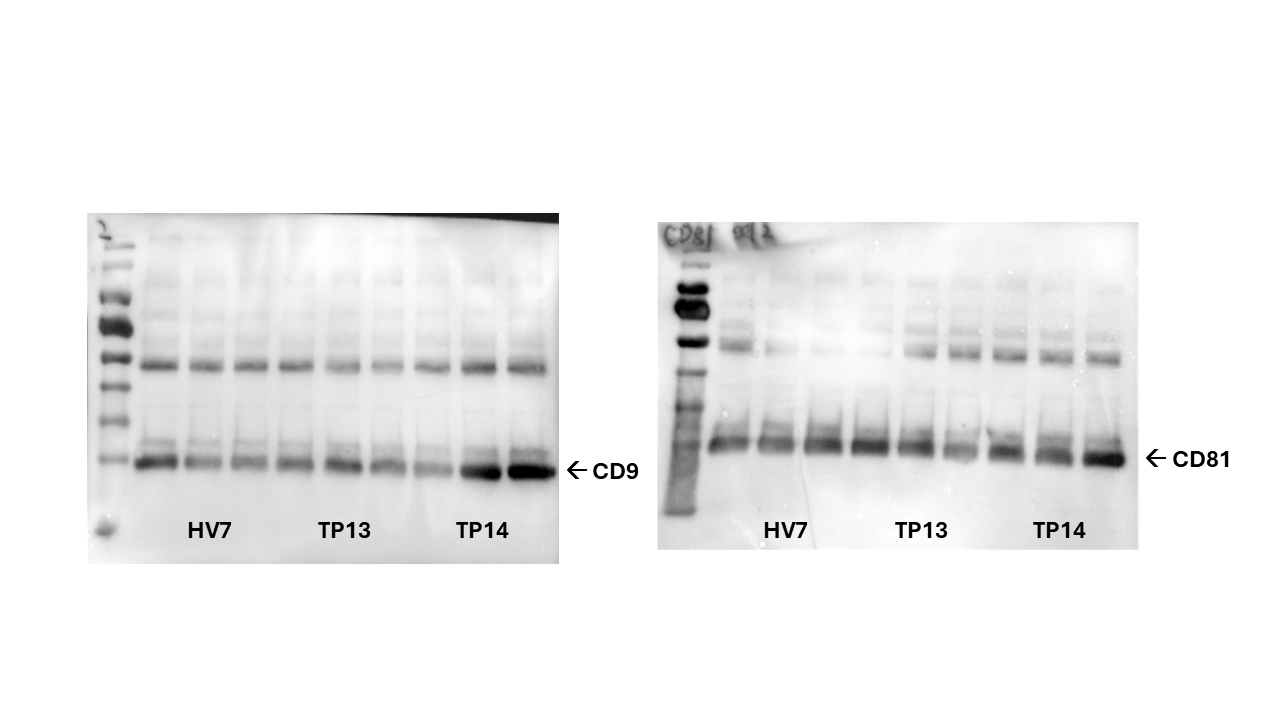

Supplement: Supplementary file 1 — Supplementary Material 1 [file 68_2026_3280_MOESM1_ESM.tif]

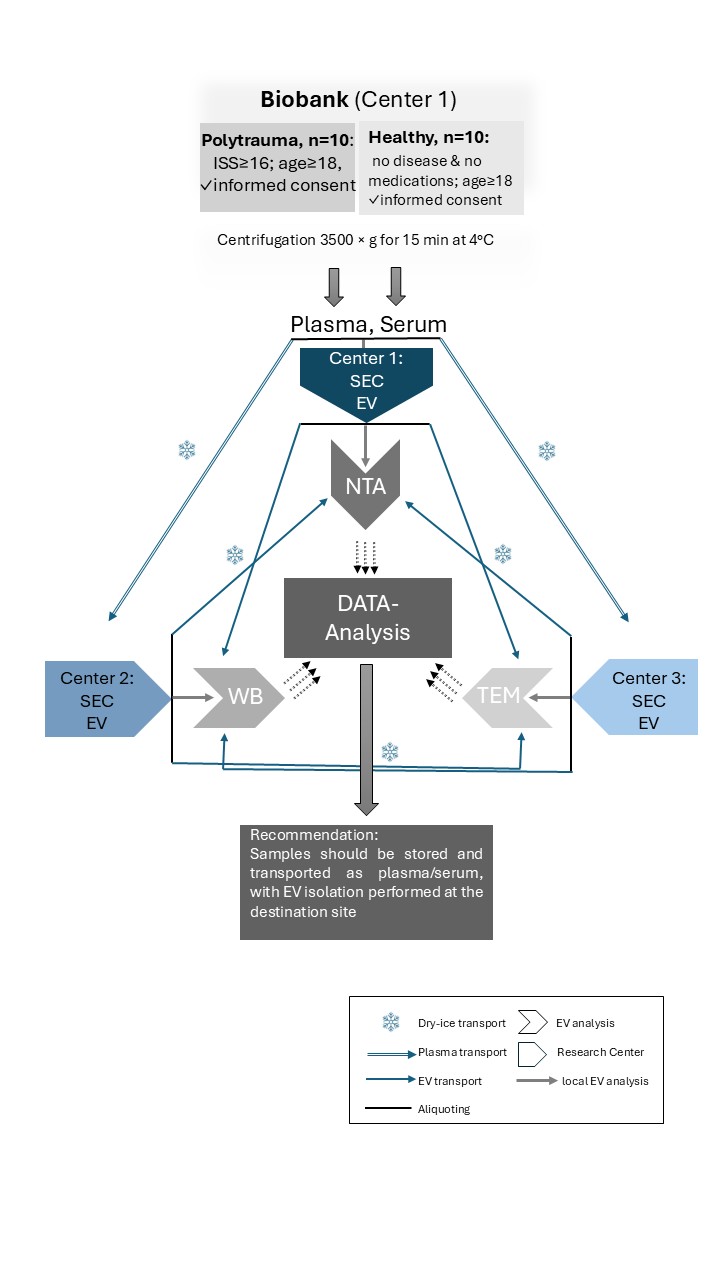

Supplement: Supplementary file 2 — Supplementary Material 2 [file 68_2026_3280_MOESM2_ESM.jpg]
